# Annotations

**Published:** 1890-11-15

**Authors:** 


### Annotations.

**Koch's Cures and other People's.**

Some of our daily contemporaries inform the public that Koch has had a considerable number of consumptive patients under his care, and has been successful with some of them. This fact is set forth in the best eloquence of the writers of sensational paragraphs as if it were something new. Of course it is natural that experiments such as Koch's should arouse some excitement in the general mind, and this we would not suppress if we could. On the contrary, we are glad of any legitimate cause which directs the attention of the larger world to the affairs of medicine. But whilst we are willing and anxious that Koch and German medicine should have all the honour which is their just due, we are not at all willing that that honour should be magnified by the unjust depreciation of the medicine of this and other countries. With regard to the cure of consumption, how does the matter stand at the present moment in Great Britain? Have any cures been effected, and in what proportion? Tens of thousands of consumptives have been cured by British physicians during the last three decades. There is hardly an intelligent village practitioner of ten years' standing anywhere who cannot record cures of his own. The truth is that certain classes of cases of true phthisis are eminently curable under favourable hygienic circumstances and in skilled medical hands. The class of cases that other physicians have cured Koch may, and probably will, cure. But he will deserve no more honour for this than thousands of other men have already deserved. What we want to see, and have a right to look for, after such an European flourish of trumpets as we have been treated to during the last few weeks, is that he shall cure cases of an advanced kind, such as other people have not cured. If Koch does this, and does it so definitely and certainly as to convince all reasonable minds, both lay and medical, we shall be among the first to vote him a statue and a place in the grand Walhalla of the heroic benefactors of mankind. But he must do, as well as reason. It is a case for works, not for faith only.

**Medical Racks and Thumbscrews.**

We have often called attention in these pages to the extraordinary degree of what may be called "irregular development" which is characteristic of the medical profession. At one end of the scale are men of philosophic mind and liberal culture, who are as competent to form judgments on all sorts of questions as leading statesmen, lawyers, or men of business. To them what is unhappily called "professional etiquette" means one thing, and one thing only—the doing unto others, on all occasions, exactly as they would be done by. Such a principle enshrined in the heart insures always both propriety and excellence of conduct. At the other end of the scale, and at all sorts of expected and unexpected points between the two extremes, are men of a very different order. They are to be found among hospital physicians and surgeons, and still more frequently among the proprietors of sixpenny dispensaries. These are the persons who, for the most part, have failed in their professional career, and who refuse to accept for themselves the responsibility of their failure. The world has been to blame in that it has been too stupid to recognise their merits; or their successful brother practitioners have been the cause, in that they have used cunning and unscrupulous arts to secure their own pre-eminence. These men leave no stone unturned to damn their more prosperous *confrères* in the eyes both of the world and of the profession. The latest phase of this malignant jealousy is that of opposition to the appearance of medical men on public platforms. A correspondent, writing to a professional contemporary, states that a friend of his, a medical man who is loved and respected by all who come in contact with him, has the fortune, or the misfortune, to be one of our best public speakers. This gentleman can make ten guineas by one evening address, and sometimes earns as much in an hour and a-half as many of his detractors do in a week. But owing to the "intolerably priggish attitude" of some of his medical neighbours, he is obliged to discontinue even his gratuitous labours on behalf of the churches, chapels, and local charities. We should have declared statements like these to be incredible if they had not been vouched for on unquestionable authority. As it is, we do not know whom to blame or pity most: the creatures who, because they cannot rise to distinction themselves, do their utmost to throw dirt on those who can; or the gifted lecturer or speaker who, having the mental capacity to instruct and charm a public audience, has not the moral courage and the Englishman's unyielding firmness to dare to defy his detractors, and to treat them with the contempt they deserve. The whole question lies in a nutshell! May the medical man claim the same reasonable freedom of speech and action as the lawyer, the clergyman, the soldier, the merchant, the tradesman, or even the agricultural labourer? Now either he may or he may not! Is there a single university, or professional college, or newspaper editor, or physician, or surgeon in any public or responsible position who will say over his own signature that a medical man may not claim precisely the same freedom of speech and action as any other man? There is not one public body or person of the kind named who will publicly take up such an untenable position. And why? Because nobody who possesses conscience and self-respect cares to expose himself to the contempt of his fellow men. The lecturer who has given up his lecturing solely on the ground of the objections of some of his brother practitioners to seeing his name on public handbills, has disgraced himself by his cowardice, and has encouraged jealous dirt-throwers of every kind to persevere in their stupid but vicious and malignant trade.

**Religion Obligé!**

A religious newspaper has at last done what we have long thought demanded imperatively to be done, and has addressed to its religious contemporaries some much-needed censures on the appearance of certain advertisements in their advertising pages. "The story is yet to be written," says our contemporary, "of the dupery of young men who have been fleeced of their money, robbed of their health, and altogether ruined by the unprincipled vultures who trade upon the fears and fatten on the moral cowardice of their victims. Were it known to what extent these unfortunate patients are bled under the threat of exposure—by men whose advertisements appear even in our religious papers and magazines—Christendom would cry shame upon a press that panders to such a trade. Is it possible that associations and societies whose organs reap profit from such advertisements are ignorant of all this? Is it a pleasant or an elevating picture to find a Y.M.C.A. appealing to its readers with a magazine thus defiled in one hand, and a pledge card of the White Cross movement in the other?" If nobility imposes obligations, and even expediency is often permitted to impose them, much more does the faith of Jesus Christ impose them, and of the highest kind. By way of justifying what we are about to say, we may inform readers that we have frequently been offered advertisements of the character under discussion, and on the comparatively unelevated ground of keeping our paper "clean and respectable" we have invariably declined them. To our thinking, lies and barely-concealed filth are as objectionable in the advertising columns of a newspaper as in its more purely literary pages. On the ground, therefore, of mere decency, all newspaper editors and proprietors who respect themselves should keep a sharp eye on their advertisement columns, and shut out filth and knavery, even at a money loss. But if this be true of mere worldly and professional newspapers, what shall be said of those that exist for the avowed purpose of teaching and spreading the Christian faith? What we say is this, that those editors and proprietors who admit advertisements some of which they know to be filthy and others which they cannot but believe to be false, either have not the intelligence to understand Christianity or have not the nobility of mind to accept it with all its obligations. The business of Christianity is to spread abroad all that is pure; this everybody will admit. It is quite as much its business to oppose the spread of all that is impure; this nobody will deny. Where then does the Christian editor or proprietor logically stand, when he not only does not oppose, but gives the fullest possible publicity to the filth of which we have spoken? He stands cap in hand at the gate of heaven asking to be admitted there at some future and convenient period, whilst he holds out behind him his other hand, into the hollow palm of which the devil drops the wages of conformity with the profitable customs of the world. All this is very edifying to the descendants of Diogenes; and no doubt equally elevating to the tastes of the members of Young Men's Christian Associations.

